# Possible ferro-electro-magnetic behaviours of graphene-based materials: hydrogenated graphene, MWCNTs and reduced graphene-oxide (GO)†

**DOI:** 10.1039/d4ra04420g

**Published:** 2024-08-20

**Authors:** Sekhar Chandra Ray, Dilip Kumar Mishra, W. F. Pong

**Affiliations:** a Department of Physics, Faculty of Engineering and Technology (ITER), Siksha ‘O’ Anusandhan Deemed to be University Bhubaneswar 751 030 Odisha India sekharchandraray@gmail.com; b Department of Physics, CSET, University of South Africa Florida Science Campus, Private Bag X6, Florida, 1710, Christiaan de Wet and Pioneer Avenue, Florida Park Johannesburg South Africa; c Department of Physics, Tamkang University Tamsui 251 Taipei Taiwan wfpong@mail.tku.edu.tw

## Abstract

This study investigated the electric polarization and magnetic behaviours of various graphene-based materials, including hydrogenated graphene (H-graphene), multi-wall carbon nanotubes (MWCNTs), and reduced graphene oxide (r-GO). Results showed that MWCNTs exhibit higher magnetization, with a magnetic squareness (*M*_r_/*M*_s_) of approximately ≈0.5, compared to H-graphene (≈0.25). H-graphene exhibits the highest electric polarization compared to MWCNTs/r-GO, whereas r-GO demonstrates the lowest levels of polarization and magnetization compared to H-graphene/MWCNTs. The valence band maximum (4.08 eV for MWCNTs, 4.26 eV for H-graphene, and 4.78 eV for r-GO) in quasi-localized states at the Fermi level results in defects in the graphene-based lattice, which are associated with dipole moment and lead to alterations in magnetic behaviours. Different density of states (DOS) is attributed from the ultra-violet photoelectron spectra and the small variations in the Fermi edge is observed in H-graphene, MWCNTs, and r-GO are responsible for the observed magnetisation and polarizations. The unique polarization/magnetization behaviours present an opportunity for potential exploitation in storage and information processing technologies in the science and engineering community.

## Abstract 2

This study investigated the electric polarization and magnetic behaviours of various graphene-based materials, including hydrogenated graphene (H-graphene), multi-wall carbon nanotubes (MWCNTs), and reduced graphene oxide (r-GO).

## Graphical abstract

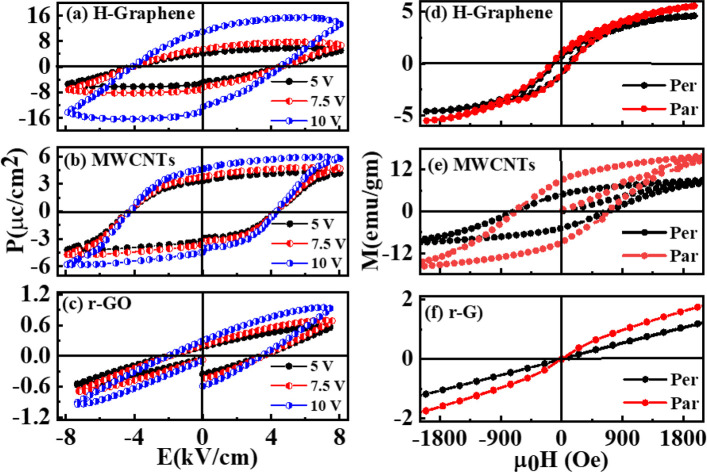

## 1. Introduction

Graphene-based materials, graphene, graphene oxide (GO)/reduced graphene-oxide (r-GO), and carbon nanotubes demonstrate excellent performance for biomedicine, biotechnology, energy and electronic applications. In biomedicine, these materials are applied for drug/gene delivery, tissue engineering, bioimaging, biosensing, and magnetic resonance imaging,^1^ whereas application in electronics include batteries, touchscreens, integrated circuits, flexible memory and solar power generation.^2^ Energy storage device graphene-supercapacitors store a large amount of energy and charge/discharge occurs rapidly, which enables superfast charging of mobile devices and fabrication of more portable high power devices.^2^ The study of ferroelectric and magnetic materials has long been a key area of focus in condensed matter physics and has resulted in a few of the most significant technological progresses to date. Local spins with off-center structural distortions are caused by magnetism and ferroelectricity.^3–6^ Displacements of ions/charge electrons are necessary for polarization charges, while magnetization requires electron spins to be ‘flipped’ without altering the position of charges. After charge separation is achieved in certain nonconducting and/or dielectric materials such as polymers and ceramics, ferroelectric properties cause electric polarization, which results in ‘electric dipole’ moment during poling/charging, whereas ferromagnetic materials are those materials that exhibit spontaneous net magnetisation, *i.e.*, the density of ‘magnetic dipole’ moments that are induced in a magnetic material (*e.g.*, Fe, Co, and Ni) at the atomic level, even in the absence of an external magnetic field. The importance of the moment of ‘electric dipole’ in ferroelectric materials lies in its use in capacitors and electrostatic interactions in various applications, such as electronics, strain sensors, robotics, thermoelectric energy generators, and batteries, whereas the use of ‘magnetic dipole’ moments in ferromagnetic materials is extensive in electrical, magnetic storage and electrochemical equipment applications. Ferroelectricity and magnetism, both in the same phase, are essential to many technological aspects. The discovery or even a weak magnetoelectric interaction, including electric polarization, in a magnetically ordered state can result in spectacular cross-coupling effects and is an exciting new development. Multiferroicity materials are mainly ferromagnetic/antiferromagnetic, ferroelectric, and ferro-elastic behaviours that could be used to fabricate logic switching and non-volatile memory storage devices.^6–8^ Thus, the research on the control of ferromagnetism by electric field becomes more prominent. Some researchers have come up with the idea of electrically controlling the magnetism of carbon-based materials through the exploration of nanomaterials.^9–12^ The present work focused on the scarcity of ferromagnetic-ferroelectric coexistence. Our study explores the ‘Ferro-Electro-Magnetic behaviors’ of recognizable dia-/ferro-magnetic graphene-based materials, *i.e.*, hydrogenated graphene, Fe-catalyst-based multiwall carbon nanotubes (MWCNT) and reduced graphene oxide (r-GO), which was influenced by their degree of graphitization. The presence of defects, density of states (DOS), adatoms, dangling bonds, and the conversion of sp^2^ → sp^3^ carbon atoms as well as the degeneracy of the π–π* band, all are crucial for magnetism, which decreases the inclination for the off-centre ferroelectric distortion. These materials have great potential in electromagnetic wave absorption because of their lightweight, high attenuation ability, large specific surface area and excellent physicochemical stability. Due to their outstanding properties of high dielectric loss, remarkable chemical stability, tremendous specific surface area and low density, these materials emerge as promising candidate for “Ferro-Electro-Magnetic” applications. It is observed that hydrogenated-graphene (Fe catalyst-based MWCNTs) has higher (lower) polarization compared to r-GO, whereas the MWCNTs (r-GO) have higher (lower) magnetization with respect to H-graphene. r-GO has lower magnetization as well as lower electric polarization. These unlikely anomalous changes in the ferroelectric/ferromagnetic behaviours of H-graphene, MWCNTs and r-GO could be the exploited. Hence, our main motivation and/or significance of this work is to exploit the magnetization as well as polarization of H-graphene, MWCNTs, and r-GO materials for storage and information processing technologies. The magnetic and electric polarization study of different morphological graphene, MWCNTs and r-GO in the same platform can produce electromagnetic waves after absorbing suitable wavelength of lights by changing the thickness of these materials. The flexible thickness and morphology of these graphene-based materials perfectly absorb the incident light of their polarization as far as possible. Through such flexible control of the thickness and perfect electromagnetic light absorption, electromagnetic waves could be controlled, which could offer potential applications in photodetectors, photovoltaics and medical diagnostics. In addition, these materials could be useful for storage and information processing devices, electronics and batteries, touchscreens, integrated circuits, flexible memory and solar power generation.^2^

## 2. Experimental details

In this study, we have used three types of graphene-based materials: (i) hydrogenated pristine bi-layer graphene thin film^13^ (H-graphene), (ii) Fe catalyst-based multiwall carbon nanotubes (MWCNTs)^14,15^ and (iii) reduced graphene oxide (r-GO).^16–18^ The bilayer graphene thin film was grown on n-type heavily doped silicon wafers (resistivity < 0.005 Ω cm) (10 mm × 10 mm) substrate by microwave plasma-enhanced chemical vapor deposition (CVD) system, equipped with a 1.5 kW, 2.45 GHz microwave source with 900 °C using CH_4_/N_2_ (gas flow ratio 1 : 4) plasma at 800 W for a duration of 60 s and then functionalized in a hydrogen plasma atmosphere^13^ at near room temperature at a chamber pressure of ∼2 torr with treatment time of 90 s and microwave power of 150 W to make a thin H-graphene film. The MWCNTs were grown by catalytic chemical vapor deposition (CVD) on silicon substrate, having dimensions of 4 × 15 cm, using carbon (camphor) and the Fe-catalyst (ferrocene) sources in 20 : 1 ratio in a pyrex flask at a temperature of 800 °C^14,15^ in nitrogen atmosphere with a deposition rate of 0.5 μm s^−1^. On the other hand, r-GO was synthesized by the H_2_O_2_ chemical reduction using graphene oxide (GO)^16–18^ synthesized by modified Hummers' method with a requisite amount of graphite powder, sodium nitrate and sulphuric acid. The surface morphology and microstructures of H-graphene, MWCNTs, and r-GO were studied using scanning electron microscopy (SEM) and Raman spectroscopy. Electron emission study was performed with electron field emission (EFE) measurements using a Keithley power supply. The electronic and bonding properties were studied by X-ray photoelectron/ultraviolet photoemission (XPS/UPS) and X-ray absorption near edge structure (XANES) spectroscopy. The C K-edge and O K-edge XANES spectra was obtained using the high-energy spherical grating monochoromator 20A-beamline at the National Synchrotron Radiation Research Center (NSRRC), Hsinchu, Taiwan. The electric polarization and magnetization were studied from the polarization (*P*) *versus* applied electric field (*E*_A_) and M–H hysteresis curves, which were measured by the ferroelectric test system (Precision LC Radiant Technology) and SQUID-type magnetometer, respectively.

## 3. Results and discussion

**Fig. 1:**
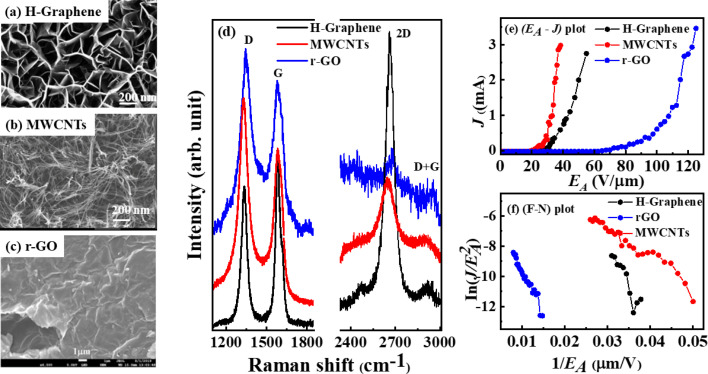
Scanning electron microscopy images of (a) H-graphene, (b) MWCNTs and (c) r-GO. (d) Raman spectroscopy of H-graphene, MWCNTs and r-GO. Electron field emission (e) *E*_A_–*J* plot of H-graphene, MWCNTs and r-GO and corresponding (f) F–N plot.

The typical SEM micrographs of H-graphene, MWCNT, and r-GO show a C=C cluster within the graphitic sp^2^-structure and are uniformly distributed throughout the surface, as shown in Fig. 1(a)–(c).^13,14,16^ The Raman spectra of H-graphene, MWCNTs and r-GO primarily show three distinct peaks: D-band is the out-of-plane breathing mode of the sp^2^-atom caused by defects, G-band is thought to represent the E_2g_ phonons at the Brillouin zone, and the 2D-band is the second order of D-band. The peaks in Fig. 1(d) are easily visible, and the degree of graphitization affects where the peaks are located. The degree of disorder is indicated by the intensity of the D peak, which is also identified as a defect-activated signature through the intervalley doubly resonance process. The *I*_D_/*I*_G_, *i.e.*, the intensity ratio of the D band (*I*_D_) and G band (*I*_D_) (tabulated in Table 1), provides information about the structural process that causes the *L*_a_ to decrease/increase. The sp^2^ crystallite size changes along with the *I*_D_/*I*_G_ ratio, indicating creation/destruction of the sp^2^ C=C bond in the graphitic structural matrix. These graphitic materials have the following ratios (*I*_D_/*I*_G_); it is evident from the values of 0.81 (H-graphene), 1.28 (MWCNTs), and 1.19 (r-GO) that MWCNTs have higher ratio, and H-graphene is graphitic in nature with the lowest ratio since H-doping promotes the three-dimensional sp^3^ bonding configuration.^19^Fig. 1(e) and (f) display the plots of the electron field emission: current density (*J*) *vs.* applied electric field (*E*_A_) and Fowler–Nordheim (F–N) plots. The *E*_TOE_ and *J* were obtained from the figures and are tabulated in Table 1. The lowest (highest) *E*_TOE_, (*E*_TOE_)_MWCNTs_[(*E*_TOE_)_r-GO_] ≈ 27.0 (52.6) V μm^−1^ and corresponding *J* ≈ 0.29 (0.001) mA cm^−2^ @ 30 V μm^−1^ strictly follow the *I*_D_/*I*_G_ ratio discussed above and degree of graphitization of these materials.

**Table 1:** The *I*_D_/*I*_G_ ratios, current density (*J*), turn on electric field (*E*_TOE_), and different electric polarization and magnetic parameters of H-graphene, MWCNTs and r-GO

	(*I*_D_/*I*_G_) ratio	*J* (mA cm^−2^) @ 30 V μm^−1^	*E*_TOE_ (V μm^−1^)	Magnetization				Electric polarization			
				Applied mag. field	*M*_s_ (emu g^−1^)	*M*_r_ (emu g^−1^)	*H*_c_ (Oe)	*V*_A_ (V)	*P*_s_ (μC cm^−2^)	*P*_r_ (μC cm^−2^)	*E*_c_ (kV cm^−1^)
H-graphene	0.81	0.06	36.3	Perpendicular	4.6	1.15	112	10, 7, 5	15.6, 7.7, 5.6	11.1, 5.4, 3.7	4.5, 4.5, 4.5
				Parallel	5.6	1.15	137				
MWCNTs	1.28	0.29	27.0	Perpendicular	11	5	693	10, 7, 5	5.8, 4.7, 4.1	4.6, 3.8, 3.2	4.4, 4.4, 4.4
				Parallel	18	9	693				
r-GO	1.19	0.001	52.6	Perpendicular	4	—	46	10, 7, 5	1.0, 0.7, 0.5	0.31, 0.21, 0.16	4.0, 4.0, 4.0
				Parallel	6	—	46				

**Fig. 2:**
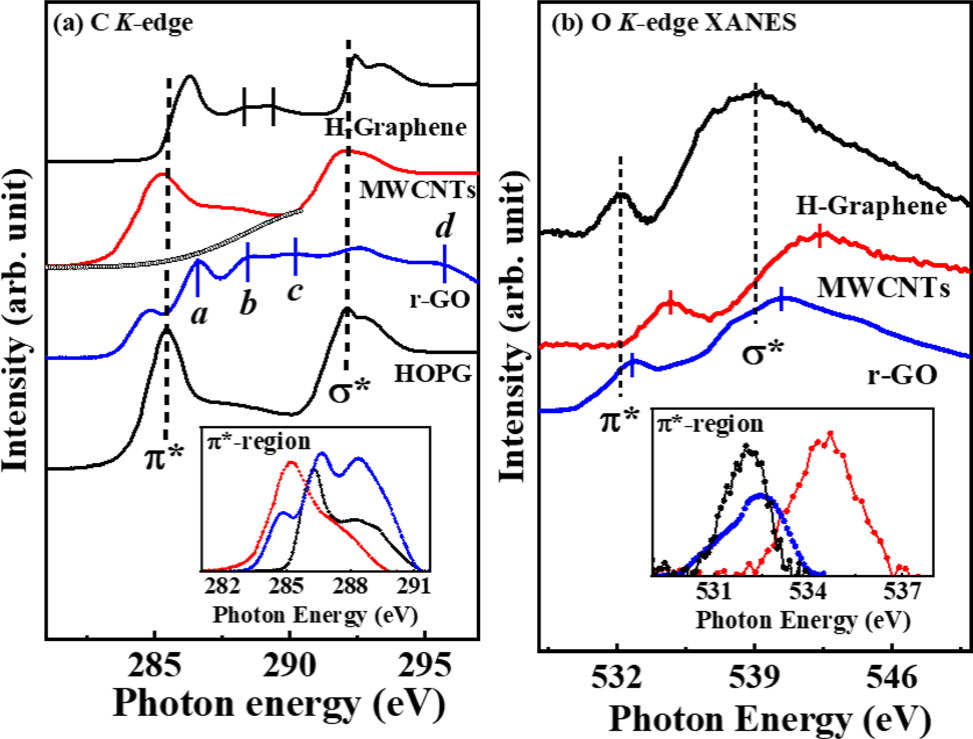
X-ray absorption near edge structure (XANES) spectroscopy at the (a) C K-edge and (b) O K-edge.

As observed in Fig. 2(a) and (b), the electronic/bonding structure of H-graphene, MWCNTs and r-GO were studied using C K-edge and O K-edge XANES spectra along with HOPG as the reference. The C K-edge X-ray absorption near edge structure (XANES) spectra in graphene-based materials are mainly separated into mainly three regions: the 
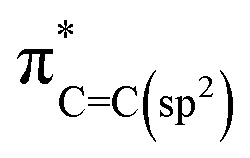
 resonance is located at ≈285 ± 1 eV, C–H* resonance is located at 288 ± 1 eV and the 290–315 eV region is associated with the 
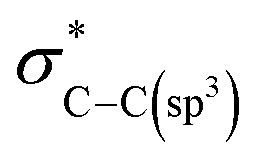
 resonance. These resonances function as the sp^2^ hybridized C–C bonds and C–H bonds fingerprint, respectively.^20^ In the C K-edge XANES, the 1s → π* and 1s → σ* state transitions of H-graphene/MWCNTs/r-GO, as shown in Fig. 2(a), could be compared to the reference HOPG, where these transitions are slightly shifted (±) with respect to the reference HOPG, from 285.5 eV → 286.3/285.3/284.8 eV and 291.1 eV → 292.4/291.1/292.6 eV, respectively, indicating that MWCNTs have higher degree of graphitization and agree well with the structural disorder observed in the Raman spectra analysis. Apart from the π* and σ* resonance peaks, a double structure wide peak within the range of 287.0–290.0 eV was observed, as indicated by the bar lines in these graphitic materials, which are known as the identifier for C–H bonds and interlayer graphite states. In r-GO, three prominent resonances are observed at ≈286.6 eV (*a*), ≈288.4 eV (*b*), ≈290.1 eV (*c*) assigned to π* (C=O/COOH), the signature of few layer graphene^21^ and π* (COOH), respectively. The resonance above σ* at ≈295.0 eV (*d*) is assigned to C=O moieties.^22^ The O K-edge XANES spectra are shown in Fig. 2(b), where the 
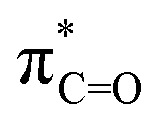
 features of H-graphene/MWCNTs/r-GO are observed at ≈532.0/534.7/532.9 eV and the 
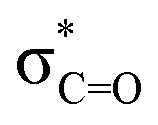
 features are observed at ≈539.3/542.7/540.5 eV.^21,23^ The π* intensities on the C K-edge (O K-edge) change as follows: 4.4 (0.87) (H-graphene) → 5.5 (1.4) (MWCNTs) → 8.38 (0.76) (r-GO). The variation in the peak position and the intensities of the π* and σ* states of H-graphene/MWCNTs/r-GO in the C K-edge and O K-edge XANES spectra indicate that C and O contents are varied without disturbing the graphitic structure. On the basis of the different microstructure/degree of graphitization, we have studied the electric polarization and compared it with the magnetic behaviours of H-graphene/MWCNTs/r-GO nanostructure materials.

**Fig. 3:**
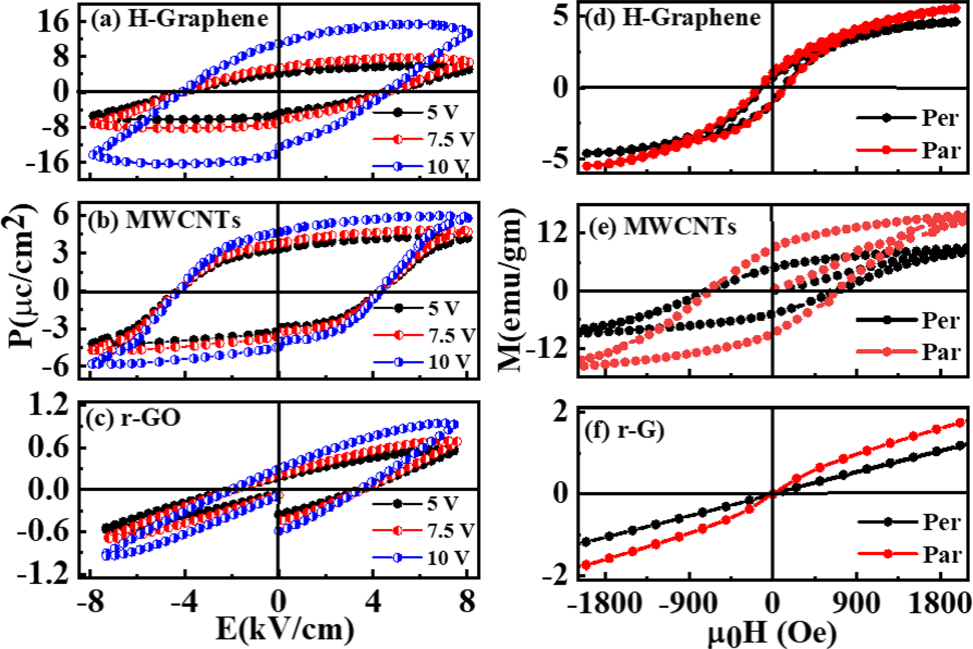
(a–c) Electric polarization of at *V*_a_ = volt of H-graphene, MWCNTs and r-GO. (d–f) Perpendicular and parallel M–H hysteresis curves (d–f) and *P*–*E* loops at room temperature for H-graphene, MWCNTs and r-GO.

Fig. 3(a)–(c) illustrates the ferroelectric behavior of the H-graphene/MWCNTs/r-GO thin films that were investigated using electric polarization (*P*) *vs.* various applied electric field (*P*–*E*_A_). All these graphene-based thin films have a ferroelectric nature, which is the mechanism of domain switching in the molecules. The saturation polarization (*P*_s_) and remanence polarization (*P*_r_) values for H-graphene were found to be higher than those of MWCNTs/r-GO, suggesting that the H-graphene content contributes more to the overall polarization because on application of an electric field, sp^2^ → sp^3^ hybridized structures that form in the atmosphere of H-plasma act as poles. In these materials, there is no significant change in the coercive field (*E*_c_). On the other hand, for MWCNTs, the internal area of the hysteresis loop is proportionally larger. This is because a leakage current would form inside the filler as a result of the overlap between MWCNTs and the creation of a local conductive network.^22,23^ The low hysteresis loss can be a result of the typical narrow-loop area ferroelectric loop of r-GO. To bring the polarization in these materials to zero, the macroscopic remnant polarization (*P*_r_) exhibited a coercive field, while the spontaneous polarization (*P*_s_) displayed a permanent electric dipole moment. The steadily increasing slope of these materials suggests that their energy storage qualities are reliable and appropriate for use in electrical energy storage devices. The increased conductivity seen in MWCNTs is the reason for the observation of a typical ferroelectric hysteresis loop as a round loop. Because of the faster domain-wall switching caused by the increased conductivity in MWCNTs, there is a large loop area and hysteresis loss. Because of their high coercivity and retentiveness, MWCNTs are therefore better suited for magnetic applications. The MWCNT reinforcement increases the piezoelectric properties and makes polling easier. However, depending on the C–C sp^2^/sp^3^ ratio, oxygen bond with carbon, defects formation, DOS, maximum valence band of the materials, the overall hysteretic behavior of polarization *versus* field dependencies varies. Fig. 3(d)–(f) shows the M–H hysteresis curve of H-graphene, MWCNTs, and r-GO measured at room temperature on applied magnetic field in perpendicular and parallel directions within the range ±2.0 kOe. The spectral features of parallel and perpendicular direction applied magnetic-field M–H loops are slightly different and is due to anisotropies in nature. The highest magnetization was found in MWCNTs compared to H-graphene and r-GO.^24,25^ The MWCNTs exhibit higher magnetization in terms of coercivity, retentivity, and saturation of magnetization compared to those of H-graphene/r-GO. Different magnetic parameters are obtained from the hysteresis loops and are tabulated in Table 1. MWCNTs have a higher magnetic squareness (*M*_r_/*M*_s_) of approximately ≈0.5, compared to H-graphene (≈0.25), whereas the squareness in r-GO is negligible. The variation of magnetization is due to their defects, DOS, degree of graphitization, and bonding structures.^26^

To support the magnetization/polarization behaviors of these graphene-based materials, XPS/UPS: He-I and He-II of H-graphene/MWCNTs/r-GO were measured and are given in S1 and shown in Fig. S1, S2(a) and (b).† The signature of the C 1s XPS spectrum is C=C/C–C, O–H/O–C–O and C=O, while O 1s spectrum is the signature of C=O, C–O, and phenolic groups, respectively.^26^ Additionally, the Fe-catalyst-based MWCNTs exhibit Fe–O/Fe–C bonds. We firmly believe that these bondings are essential to the formation of different kinds of magnetically and electrically-polarized graphene-based materials. Furthermore, as indicated in Fig. S2(a),† we estimated the VBM of these graphene-based materials from UPS measurements in He-I (*hν* = 21.22 eV) and found that the lowest ≈ 4.08 eV (highest ≈ 4.78 eV) VBM of MWCNTs (r-GO) has the highest (lowest) magnetization. A defect related to the dipole moment, which causes a change in the magnetism of the graphene-based lattice, is created by the different VBM values in the quasi-localized states at the Fermi level.^27^ The He-II (*hν* = 40.81) in Fig. S2(b)† demonstrates a subtle difference in the Fermi edge density of states (DOS) between H-graphene, MWCNT and r-GO, with the effects of its magnetism/polarizations. The valence band state's electronic structure is revealed by the UV-PES spectra, as shown in Fig. S2(b),† which shows various bonding states: C 2p_π_ (5.2 ± 0.1 eV), 2p_(π–σ)_ overlap state (7.0 ± 0.3 eV), C 2p_σ_ (8.8 ± 0.3 eV), C 2sp mixed state (11.4 ± 0.4 eV), C 2s (13 eV) and O 2s (>16 eV).^27–29^ The polarization and magnetization of these materials are also caused by these distinct π and σ bonds, which originate from C=O and the O-lone pair bonds , respectively. MWCNTs is the highest magnetization, whereas r-GO and H-graphene exhibit the less saturation of magnetization and more confined hysteretic features. These graphene-based materials display a magnetic hysteresis loop characteristic and saturate in a field of approximately ±2 kOe. This is caused by defects and various DOS and show the existence of an ordered magnetic structure. Table 1 lists the different magnetic parameters that were obtained from the loops. Due to the contiguity of Fe–C/Fe–O bonds and the ‘Fe’ catalyst ferrite phase in the structural matrix, MWCNTs exhibit higher coercive field (*H*_r_), remanent (*M*_r_) and saturation (*M*_s_) magnetization. Furthermore, in addition to bonds and ‘Fe’ catalyst, the magnetic behaviours of MWCNTs also result in other unbalanced antiparallel spins, which generate net spins distinct from those caused by structural distortion. The unpaired electrons from the defects induced by hydrogenation and the free spins available *via* the conversion of sp^2^ → sp^3^ hybridized structures are likely mechanisms for the observed ferromagnetic orders. In comparison to H-graphene/MWCNTs, r-GO has a lower saturation magnetization. The presence of different nonmagnetic oxygen/hydroxyl ions in r-GO is the cause of the lower magnetization because they suppress the magnetic behaviour. It is notable that r-GO has less electric polarization than H-graphene/MWCNTs. The higher magnetization in MWCNTs (H-graphene) is implied by their magnetic squareness of ≈0.50 (0.25) and is negligible in r-GO. When comparing H-graphene and MWCNTs/r-GO, the former exhibits the highest electric polarization, while the latter exhibits the lowest electric polarization and magnetization. The magnetic proximity effect can induce a spin-dependent exchange shift in the band structure of these graphene-based materials that could produce a magnetization and a spin polarization of the electron/hole carriers in this material, paving the way for its use as an active component in spintronic devices.

From the results discussed above, it could be clearly stated from the structural point of view that these materials are electrically polarized and ferromagnetic in nature. Thus, the charge carrier modulation of these materials using the ferroelectricity of a nearby dielectric can be useful for controlling the electronic properties of these H-graphene/MWCNTs/r-GO materials. In other ways, it also can be noted that the ferroelectric behaviours and electric polarizations of these graphene-based materials are highly corelated with their electronic structures. However, ferroelectric materials are normally in single crystalline or polycrystalline form and possess a reversible spontaneous polarization over a certain temperature range. When these graphene-based materials are located on ferroelectric oxides, their electrical coupling frequently shows abnormal behaviours, such as anti-hysteresis and in field-effect transistor operation. However, these ferroelectric graphene-based materials could possess an electrically switchable spontaneous polarization, generate broad interest because of potential applications in non-volatile memory, field-effect transistors, and photovoltaics. Without being affected by anomalous and unique polarization/magnetization behaviors of our H-graphene/MWCNTs/r-GO materials, they might be investigated for use in information processing and storage technologies.

## 4. Conclusions

The study focused on the electric polarization and magnetic behaviours of graphene-based materials, including H-graphene, Fe-catalyst-based MWCNTs, and r-GO. Without destroying the graphitic structure, each individual structure is preserved in its corresponding graphene-based structure. The nonpolar building blocks of graphene-based H-graphene, MWCNT and r-GO materials are shown to simultaneously exhibit electric polarization and magnetization at room temperature. We attribute the gating-induced interlayer charge transfer and the ability of the electron spins to be ‘flipped’ without changing their position. Despite the semi-metallicity and the nonpolar nature of the bilayer stacking-induced polarization/magnetization effects in asymmetric graphene-based materials, polarization and magnetization have been controlled *via* an external potential and magnetic field. The H-graphene shows that multiple meta-stable interlayer stacking sequences that may permit even greater electrical polarization can be obtained only by the lateral shifts of constituent monolayers. The presence of ferrite ‘Fe’ catalyst and Fe–O/Fe–C bonds in MWCNT results in higher magnetization, while the anomalous electric polarization in r-GO graphene structure is caused by the presence of the OH radical and O-related bonding. These pure graphene-based materials are non-toxic and have intrinsic magnetism associated with p-electrons in open-shell π-conjugated systems. Chemical design provides atomically-precise control of the π-electron cloud, which makes them promising for nanoscale magnetic devices. The three graphene-based materials that we studied are partially chemically designed except the H-graphene, which is highly pristine 2D graphene. In MWCNTs, mainly the magnetism comes from graphene-coated ‘Fe’-nanoparticle catalyst, whereas in r-GO, ‘OH’ radical and/or different O-related bonding is attached. Most of the researchers used compositing carbon materials with other components to overcome the limitation of attenuation mechanisms and impedance mismatch.^30^ In this work, we have studied the magnetization as well as electric polarization of three different morphology-based graphene/graphene-based materials, *viz.*, (i) pristine graphene: pure carbon atom, (ii) MWCNTs: Fe ions-based carbon and (iii) r-GO: OH radical/O bond-related carbon. Within the wide range of carbon-based materials, pristine graphene is among the most interesting for potential applications due to its intrinsic length, which facilitates contact and integration into device structures. Thus, our approach is unique and different from others. However, the polarization/magnetization behaviors of these graphene-based materials are unique but anomalous in nature, which might be useful in future information processing and storage technologies.

## Data availability

The data that support the findings of this study are available from the corresponding author upon reasonable request.

## Conflicts of interest

The authors declare no competing financial interests and have no conflicts of interest in this work.

## Supplementary Material

RA-014-D4RA04420G-s001
