# Supplementary material for: Possible ferro-electro-magnetic behaviours of graphene-based materials: hydrogenated graphene, MWCNTs and reduced graphene-oxide (GO)
Source: RSC Adv. 2024 Aug 20;14(36):26302–7. doi: 10.1039/d4ra04420g (PMC11334152; doi:10.1039/d4ra04420g)
Supplement: RA-014-D4RA04420G-s001 [file RA-014-D4RA04420G-s001.pdf]

Supplementary Information (SI) for RSC Advances.
This journal is © The Royal Society of Chemistry 2024

# Supplimentary

**S1:** XPS is a powerful method to study the bonding inside the pristine graphene, MWCNTs and r-GO. The measurements were obtained by using high-resolution XPS of C 1*s* and O 1*s* spectra. The signature of C 1*s* spectrum are C=C/C-C, O–H/O–C–O and C=O[1-9]; whereas O 1*s* spectrum is signature of C=O (oxygen doubly bonded to aromatic carbon), C-O (oxygen singly bonded to aliphatic carbon), and phenolic (oxygen singly bonded to aromatic carbon) groups respectively.[4]

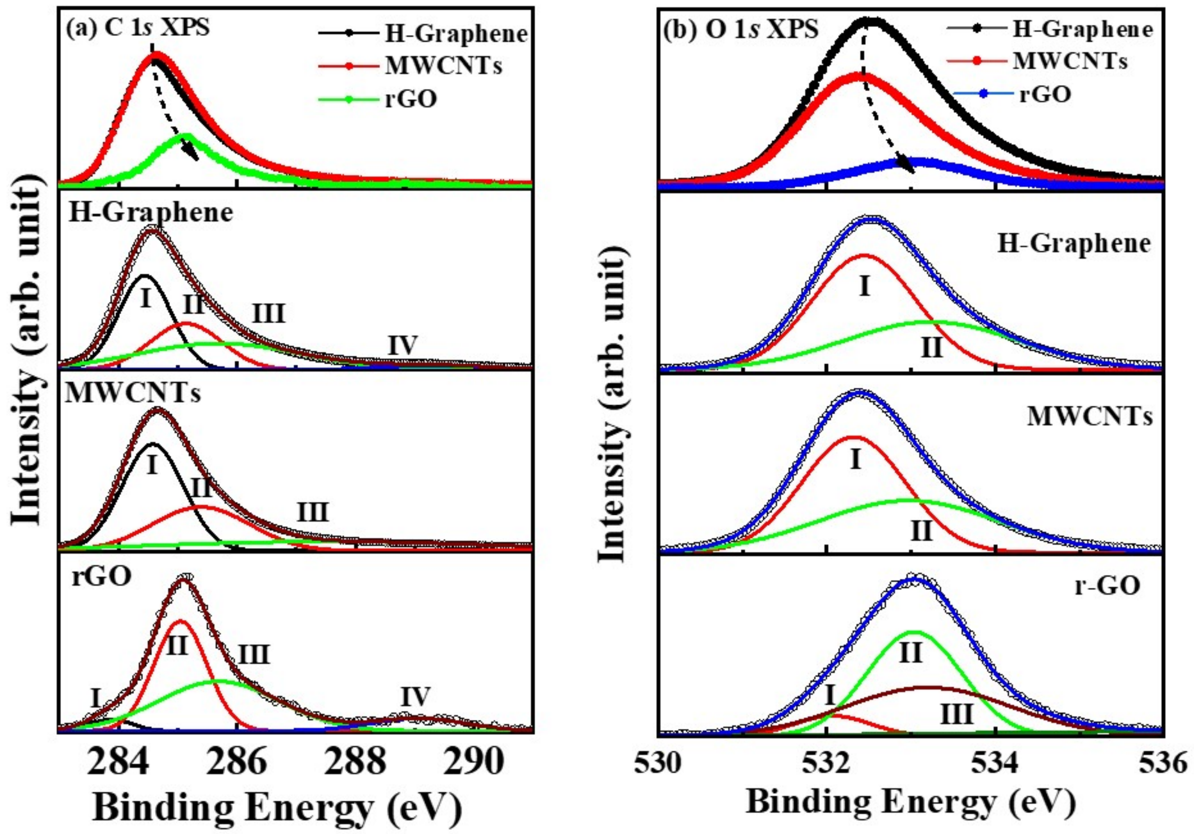


**Figure S1** (a) C 1*s* and (b) O 1*s* X-ray photoelectron spectroscopy spectra of the pristine graphene, MWCNTs and r-GO respectively and their corresponding deconvolution into different bonding structures.

Figure S1 (a, b) shows the C 1*s* and O 1*s* XPS spectra of the H-graphene, MWCNTs and r-GO respectively and their corresponding deconvolution into different bonding structures. After deconvolution, all the parameters *i.e.* their peak positions, intensity, and full-width-half

maximum are tabulated in Table S1(a). Prior to deconvolution, Shirley's background subtraction was carried out. This was done due to the inelastic scattering events the photoelectron undergoes as they are transported from the point of excitation to the surface of the sample, thus giving rise to the inelastic background intensity. This inelastic background intensity must be subtracted to determine the areas of individual peaks. In the fitting procedure, were fitted using a Gaussian curve and Lorentzian model. The C 1*s* deconvoluted to reveal the C atom bonded to oxygen functional groups, which mainly consists of C–C=C, C–OH, C=O and O=C–OH at 284.5(±0.2) eV, 286.4(±0.2) eV, 287.8(±0.2) eV, and 288.9(±0.2) eV, respectively.[1-9] Referring to Figure S1(a, b), the C 1*s* spectrum consists of peaks that correspond to $sp^2$ C=C around ≈284.5 eV(±0.3), O–H/O–C–O around ≈286.4(±0.2) eV, and C=O around ≈288.4 eV.[1-5] All the spectrum show a dominant peak around 284.5(±0.2) eV. In O 1*s* the peaks of r-GO are at ~531.3 eV, 531.9 eV and 532.3eV that are assigned to C=O (oxygen doubly bonded to aromatic carbon), C-O (oxygen singly bonded to aliphatic carbon), and phenolic (oxygen singly bonded to aromatic carbon) groups respectively.[3]

To obtain more information H-graphene/MWCNTs/r-GO, we studied the VB DOS near $E_F$ by carrying UPS measurements at He-I ($hv$ = 21.22 eV) and He-II ($hv$ = 40.81) radiation as shown in Figure S2(a,b) respectively. We have estimated valence band maximum (VBM) from the intersection of the slope of each spectrum at the higher K.E. side of UPS He-I spectra as shown in Figure S2(a) and their values are tabulated in Table S1(b). It is found that the VBM values are as follows: VBM ⇒ 4.26 eV (H-graphene), 4.08 eV (MWCNTs) and 4.78 eV (r-GO). The different VBM values in the quasi localized states at the Fermi level creates defect in graphene-based lattice that causes change in their magnetism.[54] From Figure

S2(b), a small variations of fermi edge indicates different DOS of pristine graphene, MWCNTs and r-GO that effect their magnetism/polarizations as shown in Figure 3(a,b).

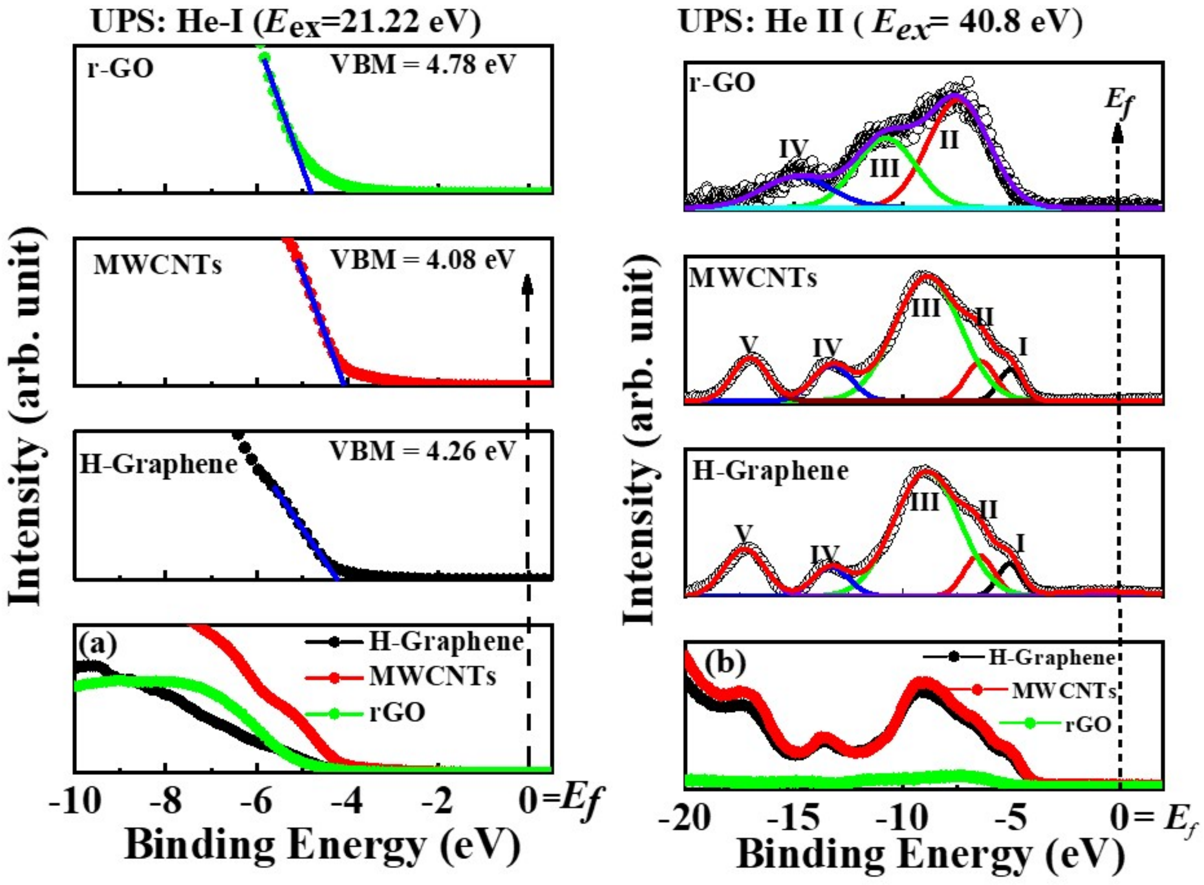


**Figure S2** (a) Valance band maximum (VBM) estimated from UPS He-I ($E_{ex}$=21.22 eV) and (b) VB-PES study from UPS He-II ($E_{ex}$=40.08 eV) spectra of H-graphene, MWCNTs and r-GO.

Higher DOS means the $sp^2$ C-C cluster along different C-/O- related bonds as discussed in XPS results (see Figure S1). Variation of VBM is related to the dipole moment. The UPS spectra corresponding to the VB DOS of pristine graphene, MWCNTs and r-GO obtained at He-II radiation are shown in Figure S2(b). We have deconvoluted the spectra into different Gaussian lines for pristine graphene, MWCNTs and r-GO within the binding energy range ≈ 0–20 eV[56] as shown in Figure S2(b). The deconvoluted Gaussian lines of pristine graphene, MWCNTs and r-GO are assigned as the C-2$p_\pi$ (5.2 ± 0.1 eV), 2$p_{(\pi\text{-}\sigma)}$ overlap state (7.0 ± 0.3 eV), C-2$p_\sigma$ (8.8 ± 0.3 eV), C-2sp mixed state ((11.4 ± 0.4 eV), C-2s (13 eV) and O 2s (>16

eV).[57-59] However, some researchers have reported that the peaks at ~13.25 eV and at 11.78 eV are due to σ and π bonds arising from C=O and the O-lone pair bonds, respectively.[60,61] Deconvoluted different parameters are also tabulated in Table S1(b).

**References**


(1) S.J. Wang, Y. Geng, Q. Zheng, J.-K. Kim, Fabrication of highly conducting and transparent graphene films, Carbon 48 (2010) 1815–1823.

(2) D. Yang, A. Velamakanni, G. Bozoklu, S. Park, M. Stoller, R.D. Piner, S. Stankovich, I. Jung, D.A. Field, C.A. Ventrice Jr., R.S. Ruoff, Chemical analysis of graphene oxide films after heat and chemical treatments by X-ray photoelectron and micro-Raman spectroscopy, Carbon 47 (2009) 145–152.

(3) J. Shen, T. Li, Y. Long, M. Shi, N. Li, M. Ye, One-step solid state preparation of reduced graphene oxide, Carbon 50 (2012) 2134–2140

(4) C. Kozlowski, P.M.A. Sherwood, X-Ray photoelectron-spectroscopic studies of carbon-fibre surfaces, J. Chem. Soc. Faraday Trans. 1 (80) (1985) 2745–2756.

(5) A. Ganguly, S. Sharma, P. Papakonstantinou, J. Hamilton, Probing the thermal deoxygenation of graphene oxide using high-resolution in situ X-ray-based spectroscopies, J. Phys. Chem. C 115 (2011) 17009–17019.

(6) H.E. Szwarckopf, B. Rousseau, Photoelectron core level spectroscopy study of Cs-graphite intercalation compounds-I. Clean surfaces study, J. Phys. Chem. Solids 53 (1992) 419–436.

(7) R. Haerle, E. Riedo, A. Pasquarello, A. Baldereschi, $sp^2/sp^3$ hybridization ratio in amorphous carbon from C 1s core-level shifts: X-ray photoelectron spectroscopy and first-principles calculation, Phy, Rev. B., 35 45101 (2001) 1–9.

(8) J.F. Moulder, W.F. Stickle, P.E. Sobol, K.D. Bomben, Handbook of X-ray photo-electron spectroscopy: a reference book of standard spectra for identification and interpretation of XPS data, Physical Electronics (1995) (Eden Prairie, MN).

(9) A.J.R. Silva, J.C. Faria, E.Z. Silva, A. Fazzio, Technical Proceedings of the Nanotechnology Conference and Trade Show, 3 (2003), pp. 165–168.

**Table S1 (a)** Deconvoluted different parameters *i.e.* the peak positions, intensity, full-width-half maximum of C 1s and O 1s spectra of H-graphene, MWCNTs and r-GO are tabulated in S1(a).

| **C 1*s* XPS study** | | | | | | | | | | | | | **O 1*s* XPS study** | | | | | |
|---|---|---|---|---|---|---|---|---|---|---|---|---|---|---|---|---|---|---|
| | **Peak I** | | | **Peak II** | | | **Peak III** | | | **Peak IV** | | | **Peak I** | | | **Peak II /Peak III** | | |
| | x (eV) | a*$10^5$ (au) | w (eV) | x (eV) | a (au) | w (eV) | x (eV) | a (au) | w (eV) | x (eV) | a (au) | w (eV) | x (eV) | a *$10^5$ (au) | w (eV) | x (eV) | a *$10^5$ (au) | w (eV) |
| **Graphene** | 284.4 | 1.3 | 1.0 | 285.1 | 0.8 | 1.4 | 285.7 | 3.2 | 2.2 | 289.4 | 0.1 | 2.2 | 532.4 | 1.07 | 1.24 | 533.2 | 0.75 | 2.07 |
| **MWCNTs** | 284.6 | 1.7 | 1.2 | 285.4 | 1.1 | 1.9 | -- | -- | -- | 287.5 | 0.8 | 6.7 | 532.3 | 0.69 | 1.23 | 533.0 | 0.54 | 2.13 |
| **rGO** | 283.9 | 0.04 | 0.8 | 285.0 | 0.5 | 1.0 | 285.7 | 0.6 | 2.4 | 289.0 | 0.1 | 1.9 | 532.1 | 0.02 | 0.90 | 533.0<br>533.2 | 0.13<br>0.11 | 1.12<br>1.97 |

**Table S1 (b)** Deconvoluted different parameters *i.e.* the peak positions, intensity, full-width-half maximum of UPS He-I and UPS He-II spectra of H-graphene, MWCNTs and r-GO are tabulated in S1(b).

| **UPS (He II) study** | | | | | | | | | | | | | | | | **UPS (He I) study** |
|---|---|---|---|---|---|---|---|---|---|---|---|---|---|---|---|---|
| | **Peak I** | | | **Peak II** | | | **Peak III** | | | **Peak IV** | | | **Peak V** | | | **VBM (eV)** |
| | | | | | | | | | | | | | | | | |
| **Graphene** | 5.0 | 0.07 | 1.3 | 6.5 | 0.10 | 1.6 | 8.9 | 0.69 | 3.6 | 13.3 | 0.09 | 1.9 | 17.3 | 0.16 | 2.2 | 4.26 |
| **MWCNTs** | 5.0 | 0.08 | 1.3 | 6.3 | 0.12 | 1.6 | 8.8 | 0.83 | 3.6 | 13.2 | 0.14 | 2.1 | 17.0 | 0.15 | 1.9 | 4.08 |
| **rGO** | -- | -- | -- | 7.4 | 0.08 | 3.3 | 10.8 | 0.05 | 3.3 | 14.8 | 0.03 | 3.7 | -- | -- | -- | 4.78 |
